# Endophthalmitis caused by *Enterococcus faecalis*: cytological and bacteriological diagnosis using vitreous fluid

**DOI:** 10.1186/s12348-025-00482-w

**Published:** 2025-03-08

**Authors:** Prithvi Ramtohul, Diane Frankel, Frédérique Gouriet, Charles Delaporte, Thierry David, Patrice Roll, Elise Kaspi

**Affiliations:** 1 Department of Ophthalmology, Hospital Nord, Aix-Marseille University, Chemin des Bourrely, Marseille, France; 2 Aix Marseille Univ, APHM, INSERM, MMG, Timone Hospital, Cell Biology Department, Marseille, France; 3 Aix-Marseille University, AP-HM, MEPHI, Marseille, France; 4 IHU Méditerranée Infection, Marseille, France

**Keywords:** Endophthalmitis, *Enterococcus faecalis*, Vitreous fluid analysis

## Abstract

**Background:**

This case report aims to describe the diagnostic method and the clinical course of endophthalmitis caused by *Enterococcus faecalis*.

**Case presentation:**

A 66-year-old man presented with sudden, severe pain and acute vision loss in his left eye. Ocular examination revealed significant intraocular inflammation and dense vitritis, suggesting endogenous endophthalmitis. Systemic evaluation identified colonic diverticulosis as a potential source of infection. Pars plana vitrectomy was performed, and analysis of the vitreous fluid, including cytological and bacteriological studies, confirmed the presence of *Enterococcus faecalis*.

**Conclusion:**

The vitreous fluid is an adequate sample for characterizing endophthalmitis by combining cytological and bacteriological analyses.

## Introduction

Endophthalmitis is a severe inflammatory condition of the interior of the eye, predominantly affecting the vitreous body and retina. This condition can arise from bacterial, viral, or fungal infections and is typically characterized by symptoms such as ocular pain, blurred vision, conjunctival redness, and, in some cases, significant visual impairment. Endophthalmitis is classified into two main categories: exogenous, which occurs following surgical procedures or trauma, and endogenous, which results from the hematogenous dissemination of pathogens from other sites within the body. Timely and effective management is essential to minimize ocular damage and preserve visual function [1–4].

Among the pathogens associated with this condition, *Enterococcus faecalis*, a Gram-positive bacterium, has emerged as a notable etiological agent, although it is infrequently highlighted in the literature. This organism, part of the normal human intestinal flora, can exhibit virulence and lead to severe complications, particularly in immunocompromised individuals or those with specific risk factors [5]. Enterococci, particularly *E. faecalis* and *E. faecium*, were reported as the third most common pathogens in health care associated infections [6], responsible for high mortality and morbidity [7]. *E. faecalis* can be responsible for both exogenous and endogenous endophthalmitis. Streptococci have been isolated from vitreous fluids following trauma, intraocular foreign bodies, or surgery (e.g., cataract surgery) [8, 9]. Other common risk factors include repeated intravitreal injections for macular degeneration treatment. In the case of endogenous endophthalmitis caused by *E. faecalis*, the primary risk factors are associated with bacteremia, such as the presence of permanent central venous catheters, illicit injectable drug use, and endocarditis [1]. Additionally, it has been reported as a complication in hemodialysis patients with catheters [4].

### Methods

Both vitreous fluid and aqueous humor were collected during the same procedure. The patient underwent a standard 25-gauge 3-port pars plana vitrectomy. Initially, a vitreous sample of 0.2–0.5 mL was obtained without infusion by gently aspirating into a syringe, resulting in an undiluted vitreous specimen. Following this, complete vitrectomy was carried out, and 360-degree laser retinopexy was performed.

The vitreous fluid sample and the aqueous humor were collected in sterile tubes.

The cytological analysis of aqueous humor and vitreous fluid was performed in the Cell Biology Laboratory (APHM). The cellularity was assessed using kova^®^ slide (Alltrista Plastics, LLC, Greer, USA). A fraction of the fresh sample was then cytospun (Thermo Electron Corporation, Cheshire, UK) at 450 g for 3 min. Slides were air dried and stained using May-Grunwald-Giemsa. Cytological analysis (Leica microscope, Wetzlar, Germany) was performed by two independent cytologists.

Bacteriological analysis was performed in Laboratoire des agents infectieux (Marseille, AP-HM) laboratory. Ten µL of the vitreous fluid sample were cultured using three different culture media: Chocolat Polyvitex (BioMérieux, Marcy L’étoile) and Columbia ANC (BioMérieux, Marcy L’étoile) media, incubated at 37 °C under 5% CO₂ for five days to grow aerobic bacteria; and 5% sheep blood Columbia agar medium (BioMérieux) incubated at 37 °C in an anaerobic incubator for 10 days to grow anaerobic bacteria. Analysis of positive bacterial cultures was performed using matrix-assisted laser desorption/ionisation time-of-flight mass spectrometry (Bruker Dlatonics, Bremen Germany). Antimicrobial susceptibility testing was performed using Vitek 2 (Bio Mérieux, Marcy L’étoile). A 16S rRNA gene amplification and sequencing was performed as previously described [10] on the anterior chamber sample.

### Case presentation

A 66-year-old man presented with sudden, severe pain and acute vision loss in his left eye, with best-corrected visual acuity reduced to light perception. He denied any history of intraocular trauma or surgery. Slit-lamp examination revealed a hypopyon, a cyclitic membrane, and vitritis, while fundus visualization was obstructed. Ultrasonography demonstrated dense vitritis, raising suspicion for endogenous endophthalmitis. A systemic evaluation identified colonic diverticulosis as a potential source. The patient subsequently underwent standard pars plana vitrectomy.

**Fig. 1:**
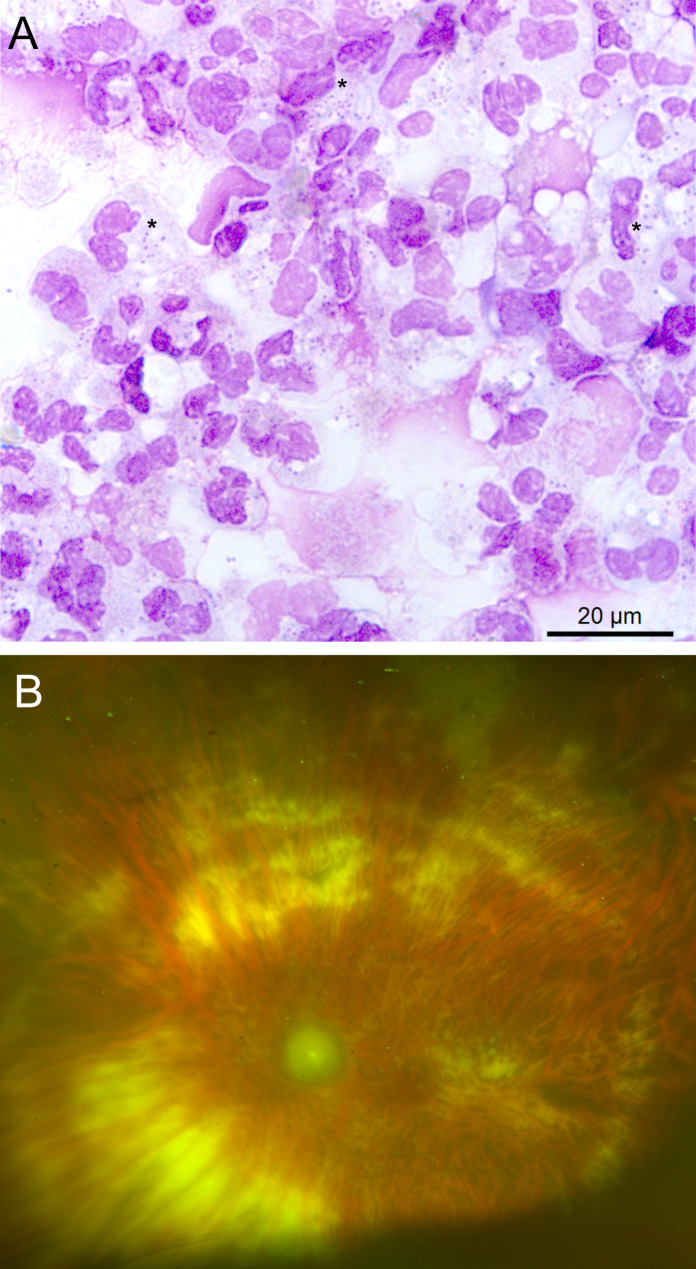
**A-** Cytological analysis of vitreous fluid showing numerous altered neutrophils and cocci, inside neutrophils (*). May-Grunwald-Giemsa staining. Scale bar = 20 μm **B-** At last follow-up, ultrawidefield fundus photography of the left eye shows complete optic disc atrophy and retinal vessel attenuation. The retina is attached with 360-degree laser retinopexy

The count of red blood cells and nucleated elements was only performed on the vitreous fluid, with the following results: 34,200 nucleated elements *per* microliter and 1,180 red blood cells *per* microliter. Conventional cytological analysis performed on both vitreous fluid and aqueous humor revealed the presence of numerous neutrophils, mostly altered, but cocci were observed only in the vitreous fluid. The bacteria were present inside the neutrophils, indicating phagocytosis, as well as in the extracellular space (Figure 1A). As the cytological analysis revealed the presence of microorganisms, the remaining fluids were transferred for bacteriological analysis.

The vitreous fluid examined by microscopy after Gram staining shows numerous Gram positive cocci. Culture identified *Enterococcus faecalis* by mass spectrometry. The *E. faecalis* was wild type phenotype, was susceptible for amoxicillin, teicoplanin and vancomycin and shows low level resistance for gentamicin [11]. The 16 S rRNA gene amplification and sequencing performed on the anterior chamber sample showed 99.9% similarity with *E. faecalis* (MF144437.1 Genbank).

The patient received systemic antibiotic therapy with amoxicillin 2 g three times daily for 15 days, in combination with 3 intravitreal injections of Ceftazidime (2.25 mg/0.1 mL) associated with Vancomycin (1.0 mg/0.1 mL) every 48 h. At the 6-month follow-up, best corrected visual acuity in the left eye remained at light perception. Ophthalmoscopy revealed optic disc and retinal atrophy (Figure 1B).

## Discussion

We present a case of endophthalmitis caused by *E. faecalis*. Notably, while the aqueous humor exhibited signs of inflammation, no microorganisms were detected using conventional cytological analysis and Gram staining. In contrast, the vitreous fluid yielded a larger volume of material. Cytological analysis identified the presence of microorganisms, and the remaining fluid was sent to the bacteriology laboratory, facilitating the identification of the pathogen and allowing for targeted treatment based on the antibiogram.

Post-cataract endophthalmitis occurs in approximately 0.1% of cases, with *Enterococcus* identified in less than 5% [1]. Furthermore, there has been an increase in endophthalmitis following intravitreal injections of anti-vascular endothelial growth factor (VEGF), commonly used to treat neovascular macular degeneration [1].

One of the largest cohort publication retrospectively recruited, during a 12-year period, 390 patients presenting endophthalmitis, with culture-proven microorganism; among them, *E. faecalis* was isolated from eye samples from 37 patients (9.5%) [5]. The infection mostly occurred following a cataract surgery (73%). The germ was almost consistently isolated from the vitreous fluid but isolation from the aqueous humor was more challenging (95% versus 51%, respectively). The antibiogram indicated susceptibility to vancomycin, penicillin, ampicillin, teicoplanin and level resistance for gentamicin; however, 27% (6/22) of cases, including one-third of those post-cataract surgery, demonstrated high-level resistance to gentamicin [5].

Additionally, a study by Kim et al. involving 174 patients with endophthalmitis reported an increased prevalence of *E. faecalis* in culture-proven infection rising to 20% (20/96) and highlighted this microorganism as one of the most common leading to reduced light perception. This study also highlighted a low rate of positive bacteriological cultures, found in only 59% of cases (103/174) [12].

In conclusion, vitreous fluid is an adequate sample for the etiological diagnosis of endophthalmitis, allowing for a combination of cytological and bacteriological analysis, thereby optimizing therapeutic management.

## Data Availability

No datasets were generated or analysed during the current study.
